# Assessing the Influence of Maternal Age in Bovine Embryos and Oocytes: A Model for Human Reproductive Aging

**DOI:** 10.14336/AD.2024.0305

**Published:** 2024-04-05

**Authors:** Aleksander Franciszek Butkiewicz, Ana Amaral, Marta Cerveira-Pinto, Pawel Kordowitzki

**Affiliations:** ^1^Department of Basic and Preclinical Sciences, Institute of Veterinary Medicine, Nicolaus Copernicus University in Toruń, Lwowska 1, 87-100 Toruń, Poland.; ^2^CIISA—Centre for Interdisciplinary Research in Animal Health, Faculty of Veterinary Medicine, University of Lisbon, 1300-477 Lisbon, Portugal.; ^3^Associate Laboratory for Animal and Veterinary Sciences (AL4AnimalS), 1300-477 Lisbon, Portugal.; ^4^Department of Gynaecology, European Competence Center for Ovarian Cancer, Charité, Berlin, Germany.

**Keywords:** cattle, oocyte, embryo, blastocyst, epigenetics, reproductive aging, woman

## Abstract

In the first weeks after fertilization, embryo mortality in cattle is significantly higher. It is well known that the age of the dam is one of the crucial factors affecting the quality of embryos and oocytes in many mammalian species. In older cattle, there are several evidences that embryo quality decreases, due to a decrease in ovarian reserve, a decrease in mtDNA and ATP, a decrease in progesterone levels, and due to susceptibility to genetic mutations. Herein, we intend to provide an updated summary of recent research on the effects of maternal age on embryos and oocytes of domestic cattle which are a widely used model species for human oocytes and early embryonic development.

## Introduction

**Figure 1.**
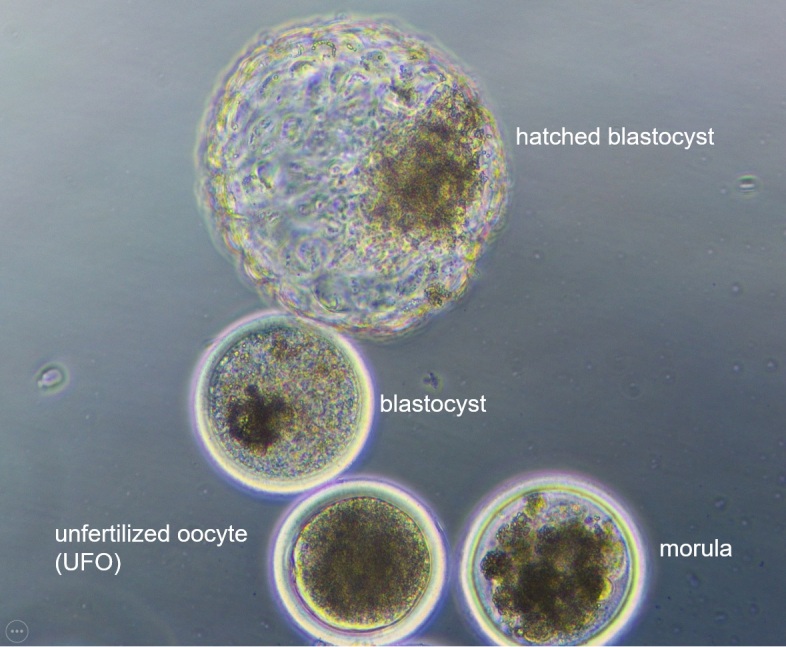
Bovine pre-implantation embryos at different stages and one unfertilized oocyte.

A major factor contributing to the low reproductive efficiency of high yielding dairy cows is high embryo mortality. A significant proportion of embryo loss in cattle, especially in lactating dairy cows, occurs even before maternal recognition of the embryo, which usually occurs around day 16 after fertilization [1]. Maternal age is one of the most important factors affecting fertility and embryo quality in numerous mammalian species. The study of oocyte aging can be furthered using various in vitro systems, also in the bovine species as a model for human oocytes. The bovine species is advantageous for controlled studies of the effects of aging and age-related insults on oocyte structure and function, as well as the effects of various treatments on aging. Such studies have practical applications in the preservation of fertility and prevention of birth defects in older women. An important part of studying oocyte aging is distinguishing changes that are an inevitable part of the aging process from changes that are due to various environmental and genetic insults and thereby determining ways to prevent or reverse certain age-related deficits. For the study of aneuploidy and other age-related meiotic errors, the cow is an adequate model system because it shares several characteristics of reproductive physiology with humans. However, a major difference between bovine and human oocyte aging is the relatively short lifespan of cattle. This is thought to be advantageous for certain types of studies, particularly those involving hormone or drug treatment at different ages. Noteworthy, the quality of oocytes is crucial for the success rate of in vivo or in vitro fertilization [2]. Embryonic development is a highly complex and sequential multi-stage process, during which the embryo undergoes rapid cellular division and differentiation. As a result, embryos are vulnerable to various external and internal factors, potentially leading to developmental abnormalities or failure. With the development of ultrasound technologies and the implementation of hormonal protocols, such as the induction and synchronization of estrus and superovulation protocols, interest in cattle reproduction increased significantly. To meet the challenges of breeding in the 1980s, veterinarians and breeders intensified the use of assisted reproductive technologies. An example of such a solution is embryo transfer, which is effectively used in cattle breeding, allowing a cow with lower genetic potential to be used as a surrogate for calves with particularly promising potential [3,4]. A second solution that arose not much later is the ovum pick-up (OPU) method based on the ultrasound-guided aspiration of immature oocytes which then can be used for the *in vitro* fertilization procedure [5]. As previously mentioned, the bovine and human reproductive systems share some similarities such as the ovarian follicular dynamics, and endocrine control closely resemble those of humans. Additionally, a large number of oocytes can be easily collected from bovine ovaries, either *in vivo* or *post mortem* since slaughterhouse ovaries are organs that are not used for human food production [6-8]. Interestingly, the characteristics of oocytes’ developmental processes are evolutionarily conserved, and phylogenetic analysis of proteins involved in the fertilization process has revealed that human oocytes are more closely related to bovine oocytes than murine oocytes in this respect [7]. Noteworthy, several factors affect oocyte and embryo quality. These factors include the nutritional status of the oocyte donor [9], environmental hygiene [10], infectious diseases [11], heat stress [12], and others. Taking care of embryo quality is of particular importance, as studies show that the highest success/pregnancy rate in cattle after embryo transfer (ET) can be reached if good quality embryos, mainly at the morula and blastocyst stage (Fig. 1), are transferred [13-16]. In this regard, maternal age is a crucial factor, influencing the ET outcome and born-offspring rate not only in cattle. Previous studies provided evidence that pregnancy rates decrease with maternal age [17-20]. This review aims to provide a summary of the effect of maternal age on the quality of bovine oocytes and embryos. and how it relates to similar aspects in humans, providing a basis for using the bovine species as a model for human reproductive aging.

## Effect of Maternal Age on Oocytes

**Figure 2.**
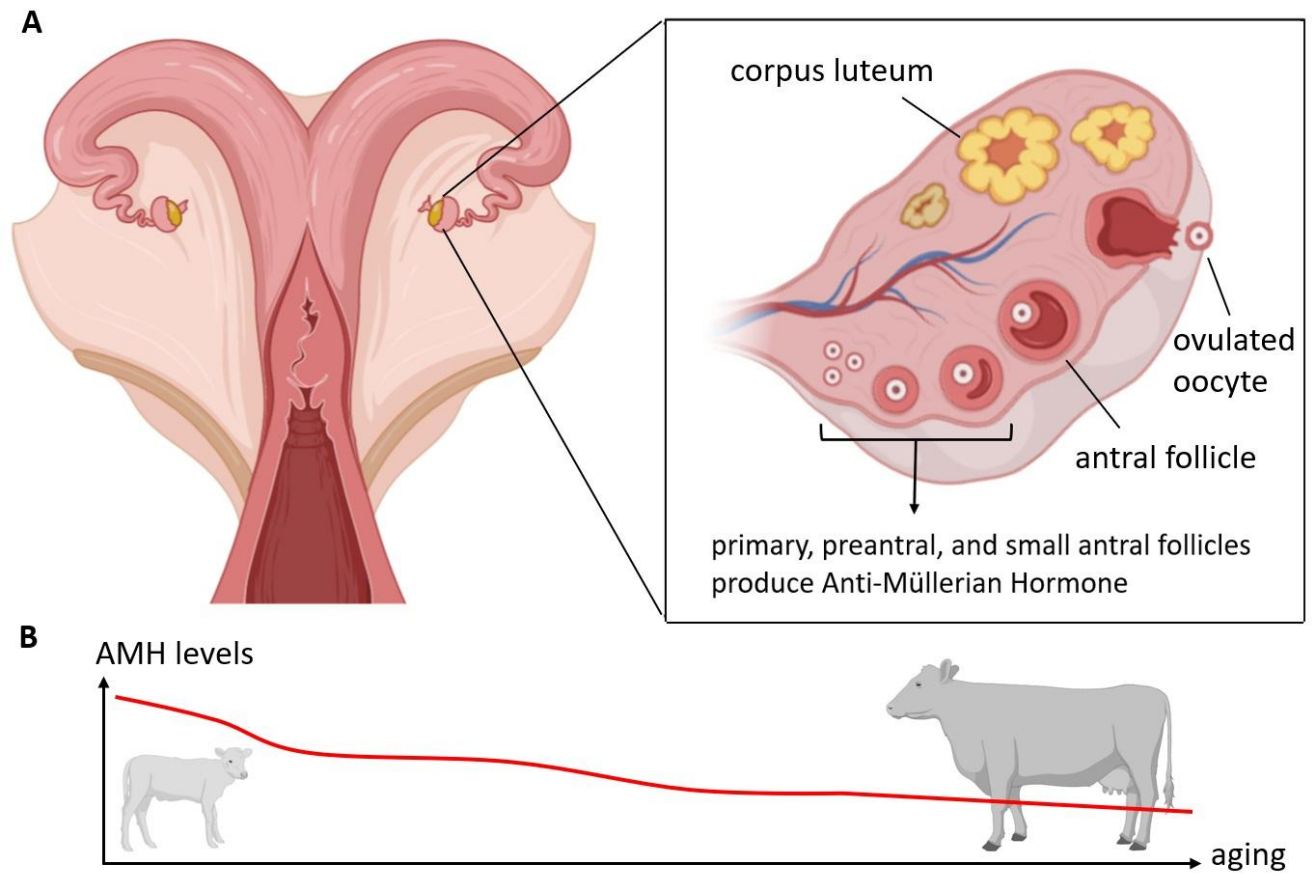
**Anti-Müllerian Hormone in the bovine reproductive tract**. (**A**) Scheme shows the bovine reproductive tract with a zoom into the ovary, its follicles at different stages, the ovulated oocyte, and the corpus luteum. The origin of the Anti-Müllerian Hormone (AMH) is also depicted. (**B**) As the number of available follicles diminishes with advancing maternal age, the AMH level decreases.

As mentioned briefly in the Introduction, maternal age affects female reproductive organs in mammals. Oocyte quality is determined by processes including oocyte maturation, division spindle formation, energy supply, syngamy, early embryonic development, and epigenetics [2, 21-23]. The number of oocytes is a crucial issue that decreases with age [24]. In ruminants, the process of ovarian follicle formation begins during fetal life, with the process of cell differentiation ending in the first trimester [25, 26]. There is a process of proliferation and differentiation into primary follicles in which the oocytes are protected [27]. In cattle, the process of primary follicle differentiation usually begins between the 90. and 140. day of fetal life [24, 28-31]. Noteworthy, the population of oogonia in the developing bovine ovary reaches a maximum estimated number of around 2.1 million (approximately at the 4.-5. months of gestational age) [25], which decreases over time and at birth is around 130,000 [24]. With each successive ovulation, the ovarian reserve decreases, moreover, atretic processes occur which reduce the potential number of ovarian follicles/oocytes (Fig. 2). It is worth highlighting the importance of research on Anti-Müllerian Hormone (AMH), which has wide application in humans. AMH was originally implicated in early sex differentiation. In male fetuses, Sertoli cells produce AMH, which facilitates the regression of Müllerian ducts, enabling the development of the male reproductive system, whereas, in females, the lack of this hormone allows the Müllerian ducts to develop into female reproductive structures [32, 33]. Additionally, in cycling females, granulosa cells of antral follicles synthesize AMH, making it a potential molecular biomarker for assessing ovarian reserve [32, 33]. However, while AMH holds promise as a biomarker in reproductive medicine, challenges persist in standardizing assays and interpreting results across different laboratories and species [34]. Further research is necessary on the practical application of AMH to thoroughly understand its value as a diagnostic marker.

In the context of oocyte quality assessment, many authors use the mouse as an animal model. Of course, this is justified to a certain extent, as the mouse is the most accessible mammalian species for laboratory studies. However, compared to humans, the reproductive life cycle of the mouse is much shorter and the mouse itself belongs to a completely different taxon [7,35]. In humans, a decline in oocyte quality is thought to be one of the most important factors contributing to fertility decline in advanced-age women [35-37]. As previous studies in cattle have clearly shown, the ability of ovarian follicles and oocytes to respond to hormonal signals decreases in parallel to increasing maternal age [2, 20, 35, 38]. In a study by Iwata et al. [39], fertilization rates of oocytes generated from older cows were investigated and revealed a correlation between increasing age and a decrease in fertilization success. The proliferative activity of granulosa cells from follicles obtained from older cows was found to be lower, and their telomerase activity and telomere length were diminished, compared to younger counterparts [40, 41]. As previously briefly mentioned, the number of atretic ovarian follicles increases with maternal age. The primary mechanism leading to ovarian follicle atresia is apoptosis, the genetically determined death of ovarian follicle cells [42]. Apoptosis starts well before any morphological signs of degeneration appear [43]. However, the induction, development, and subsequent stages of apoptosis in vesicles are still not fully understood. Zeuner et al. [42] report that atresia of ovarian follicles is relatively difficult to detect and assess but, even if detected at an early stage, can already affect the secretion of harmful metabolic products into the follicular fluid, significantly limiting the developmental competence of the oocyte [42, 44-48]. According to our experience up to 40% of immature oocytes can reach the blastocyst stage in the bovine species *in vitro*, as reported by other groups, too [49]. Older oocytes are more susceptible to the occurrence of genetic mutations and chromosomal aberrations, which increases the risk of genetic abnormalities in the offspring and miscarriages [50].

## Effect of Maternal Age on Embryo Development

To the best of the authors' knowledge, there is a paucity of scientific publications discussing the effect of cow age on embryonic development. Malhi et al. [20] conducted a study in which they collected embryos from old (13-16 year-old) and young (3-6 year-old) cows. Their results showed that maternal aging affects the decrease in fertilization rate and the cleavage of mature and fertilized embryos in vivo in cattle. Furthermore, the same authors mention that aging is reported to be associated with fewer follicles of 2-5 mm in diameter at the onset of the follicular wave and a reduced follicular and ovulatory response to hormonal therapy. In women, it has been shown that mitochondrial function at the morula stage decreases with maternal age and that it leads to a slower developmental rate and impaired development from morula to blastocyst [51-53]. Takeo et al. [7] came to a similar conclusion in their study on embryos of Japanese black cattle aged between 25 and 209 months. Younger cows were shown to have a higher ratio of embryos cleaved at the 4-cell stage. It is worth noting that the authors emphasize that the number of mitochondrial DNA (mtDNA) remains constant and does not show significant differences depending on the division status of the embryos. Another experiment compared the number of mtDNA in metaphase II (MII) stage oocytes and embryos 48 hours after insemination. Most cows exhibited a decreased number of mtDNA, although some experienced an increase, while older cows (> 180 months) generally displayed lower mtDNA levels in oocytes. The change in the number of mtDNA during early development varied between cows, although it generally decreased. In summary, the study concluded that age affects the cleavage capacity of oocytes and the number of mtDNA in oocytes varies between individuals. Furthermore, a negative relationship was observed between cow age and embryonic development. Soares et al. (2020) monitored parameters of bovine oocytes such as cytoplasmic volume, mass, distribution and aggregation of mitochondria, mitochondrial activity, and levels of mitochondrial hydrogen peroxide (H_2_O^2^). The results of this experiment indicate a significant decrease in the cytoplasmic volume of oocytes upon aging. Additionally, the level of H_2_O_2_ increased significantly due to aging, and the patterns of mitochondrial aggregation were significantly different after 30 hours of *in vitro* maturation, with MII oocytes presenting small aggregates within the cytoplasm while aging oocytes lacked granules. In contrast, there were no differences observed between different aging groups in terms of mass, distribution, and activity of mitochondria [54].

Some authors also suggest that there is a significant influence of adenosine triphosphate (ATP) on bovine embryonic development and its amount decreases with maternal age [39, 55]. The research group of Shen et al. [56] conducted a study on the genetic recombination rate in cattle. The recombination rate in genetics is a measure of the frequency of recombination, the process of shuffling and exchange of genetic material between homologous chromosomes, during the formation of reproductive cells, or gametes. The process of recombination plays a key role in shaping genetic diversity and the mechanisms of inheritance. It produces new combinations of genes and alleles, which can lead to unique genetic changes in a population. High recombination rates can increase genetic diversity in a population, while low recombination rates often maintain the genetic integrity of genes that are linked. In the study by Shen et al. [56] a lowered recombination rate was observed between 20 and 65 months of age of the dam, and then increased when the cow's age exceeded 65 months. These results were in line with the research of Martin et al. [57] in the human species. In consequence, one can generate new insights into the rate of genetic recombination, a key process affecting genetic diversity and inheritance mechanisms. Understanding this process is important since it can shed light on analogous mechanisms in humans. Analyzing such patterns in cattle can provide valuable parallels regarding reproductive mechanisms related to maternal age in humans. The study of genetic recombination rates in cattle provides us with a comparative perspective, which supports our understanding of analogous processes in humans. Furthermore, it highlights the need to consider external factors influencing genetic outcomes, which is of particular importance in studies of reproductive mechanisms related to maternal age. In humans, a significant concern is aneuploidies. The increasing trend of delaying motherhood in women leads to a constant rise in maternal age [57]. Aneuploidies in humans constitute one of the most common causes of infertility and developmental disorders in children and occur in about 0.3% of newborns, 30-60% of embryos, 30-70% of oocytes, and 35% of fetuses from spontaneous abortions [58, 59]. For decades, the correlation between maternal age and the incidence rate of trisomy 21 (Down syndrome) has been well-known [60]. The risk of having a child with trisomy 21 increases from about 3% in younger women (aged 20 years) to 30% in advanced-age women (aged 40 years) [61], based on chromosome segregation errors during meiosis I [62]. In the case of cattle, the issue is not as significant since cows reproduce at young ages. Nevertheless, cases of trisomies have been described, including trisomy 20 [63], 22 [64], 28 [65], and X [66]. In humans, chromosome segregation errors can occur both in the fetal ovary during mitotic proliferation of primordial germ cells, forming oogonia, and during meiotic divisions of oocytes, as well as during mitotic divisions of the early embryo after fertilization. This is most likely due to failures in meiotic recombination, worsening chromosome cohesion, defects associated with the karyokinetic spindle, changes related to histones and tubulins, as well as mitochondrial dysfunction [55, 67]. Studies in cattle confirm the significance of abnormal cleavage concerning genomic instability within the embryo. Interestingly, Destouni et al. (2016) reported cases in which normally fertilized eggs underwent what they termed "heterogenic divisions" — a type of cleavage, most likely trichotomous, resulting in androgenetic, gynogenetic, and biparental blastomeres [68]. Given the conservation of such mechanisms of genomic instability between bovine and human embryos, they offer insights into understanding cases of human mixoploidy and chimerism [69]. As reviewed elsewhere, the occurrence of aneuploidies in *in vitro* matured oocytes compared to *in vivo* is not straightforward [70]. Some studies suggest that there is no significant difference in the occurrence of aneuploidies between *in vitro* and *in vivo* methods [71]. Other studies indicate that the *in vitro* protocol may affect the karyokinetic spindle and increase the risk of aneuploidy [72-77]. In studies conducted by Lee et al. 2006, it was shown that patients in the younger age group had significantly higher numbers of retrieved oocytes and higher rates of pregnancy, implantation, and multiple pregnancies [78]. However, this does not mean that advanced-age women do not have a chance of having healthy offspring. With the advancement of ART, the rates of successful fertilization and implantation also increased in advanced-age women [79].

## Effect of Maternal Age on Pregnancy and Fetal Development

In a study by Kuhn et al. [80], conception rates in heifers reach a maximum outcome at 15-16 months of age. Starting to inseminate heifers at 26 months of age or later resulted in a 13% decrease in the number of pregnancies per insemination, probably due to lower embryo survival rates. Young cows, especially heifers, are believed to have a calf birth weight 10% lower compared to adult cows [81]. This may be due to differences in the energy imbalance between young and older cattle [81-86]. In studies conducted on sheep, it has been shown that still-growing mothers use nutrients for their development at the expense of the fetus [27, 87] resulting in a reduced placental surface area. In addition, placentas of heifers were found to be smaller in size than those of mature cows and also showed reduced cotyledon weight and lower total cotyledon area [88]. Therefore, it can be assumed that embryonic and fetal development in young cattle is hampered both by competition for nutrients between the mammary gland and the dam and by the growth of the dam itself. This means that a growing embryo or fetus in young cattle may encounter difficulties in obtaining sufficient nutrients for its development due to competition for the demand for milk production (by the mammary gland) and the needs of the developing offspring. Despite the low nutritional requirements of the embryo in the early stages of gestation, its metabolic activity is high, and this represents a critical period in which the development of many organs begins [89]. Among the studies supporting the thesis of a significant effect of maternal age on the number of cotyledons during pregnancy, one can also find several that contradict this [90, 91, 92]. Another important aspect is the progesterone level during pregnancy. Progesterone is a key hormone required to sustain pregnancy. It regulates both the thickness and structure of the endometrium, while also acting as a relaxant on the uterine muscles, which is an important mechanism to protect against premature contractions that can endanger pregnancy. In addition, progesterone increases blood flow to the uterus, thereby supporting the proper delivery of nutrients and oxygen to the developing fetus. This hormone also plays an important role in the development of the fetal organs and prepares the mammary glands for milk production [93]. One of the main factors leading to decreased fertility in older cows is believed to be the reduced production and secretion of progesterone, especially during the early luteal phase [94-96]. Reduced progesterone secretion during the early luteal phase also reduces the production of interferon tau, which is the embryonic signal of pregnancy in cattle [97]. Research by Parr et al. [98] suggests that if progesterone supplementation is decided upon, it should be introduced during the early luteal phase. Both in women and female cattle, the surge in LH induces ovulation, after which the formation of the corpus luteum occurs, responsible for progesterone production [99]. It is worth noting that there is a connection between the level of progesterone and the occurrence of stress. Studies have shown that this type of stress can both increase and decrease the production of this hormone [100]. In women, an early hormone that is indicative of pregnancy is the human chorionic gonadotropin (hCG). This hormone prevents the lysis of the corpus luteum, thus supporting the maintenance of early pregnancy. It is worth emphasizing that in *in vitro* studies, ovine LH has been found to increase the synthesis of progesterone in the human corpus luteum, similar to hCG [101]. The principle of hCG action is similar to bovine interferon tau, although the mechanism is different. The luteal phase in cattle includes metestrus and diestrus, lasting about 80% of the entire cycle (approximately 16 days) [102]. In women, the luteal phase lasts from 12 to 17 days [103]. Similar to cattle, women are also known to experience the stimulation of the luteal phase. Progesterone (P4) supplementation is preferred due to the lower frequency of ovarian hyperstimulation syndrome (OHSS). Its use is recommended until the pregnancy test is performed from blood serum. Various forms of progesterone medications are available, such as oral, intramuscular, and vaginal. Intramuscular and vaginal P4 compounds show comparable rates of implantation and clinical pregnancy, although higher levels of progesterone in the blood serum are achieved after the injection of the intramuscular preparation [104]. Considering the issue of aging oocytes and embryos in cattle breeding, it is crucial to take this aspect into account from a zootechnical perspective when managing the herd, too. First and foremost, controlling inbreeding (controlled mating between close relatives) is essential to prevent inbreeding depression in the herd [105]. Inbreeding depression has been proven to increase embryo mortality and the occurrence of stillborn animals. Additionally, there are fertility issues in inbred adult animals, including lack of ovulation, lower sperm quality, and quantity [106].

**Figure 3.**
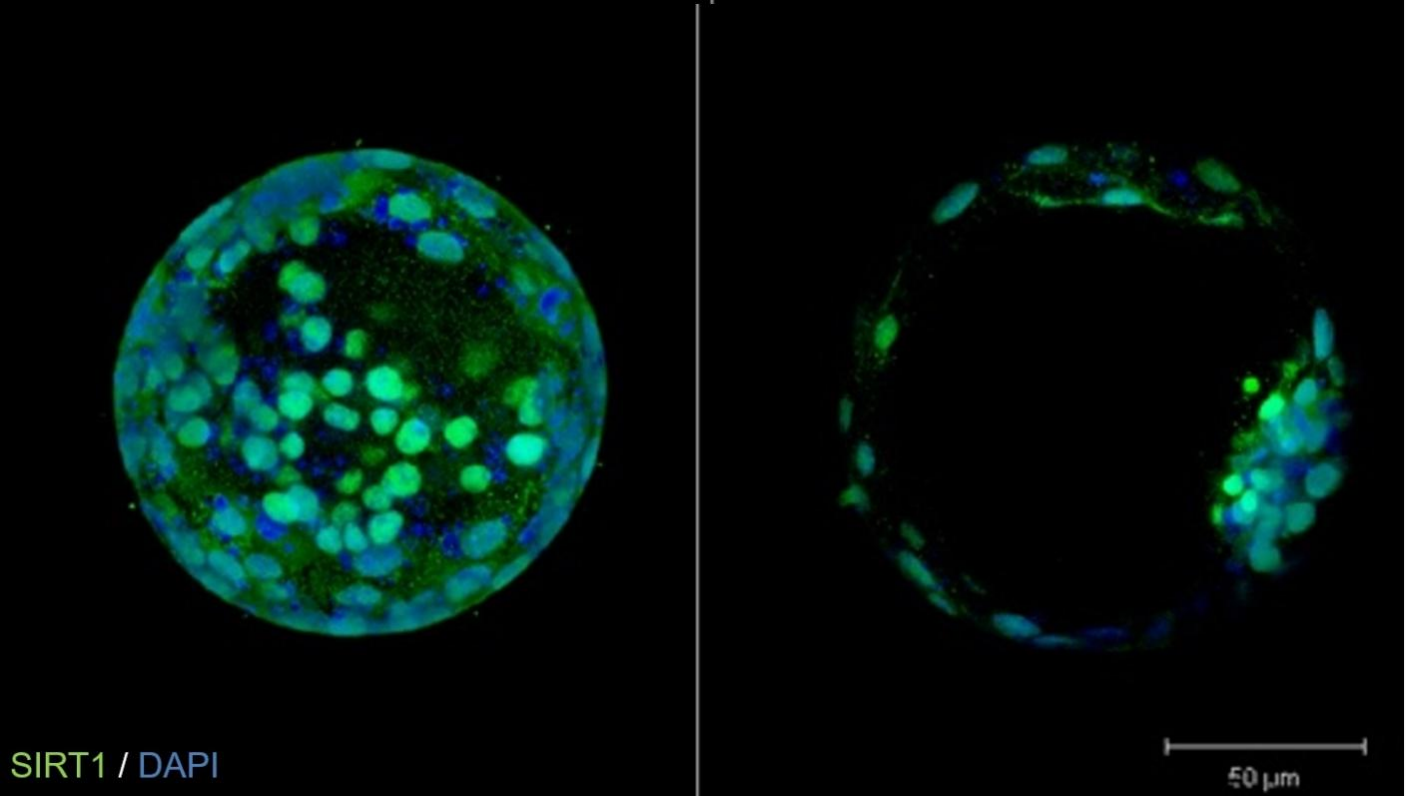
Confocal microscopy pictures show a bovine day 7 *in vitro* produced blastocyst with stained DNA (DAPI in blue), and SIRT1 protein expression (green) in two different sections.

The causes of inbreeding depression are attributed to two sources: the first involves the meeting of recessive genes in a homozygous system, allowing their expression in the phenotype. The second theory suggests that the heterozygous state is inherently more advantageous than the homozygous state. The advantage of heterozygosity is linked to greater adaptability to environmental conditions compared to individuals with lower genetic variability, which consequently results in reduced adaptive capabilities [107]. Effective herd management requires prioritizing the treatment of young animals, especially in the context of reproductive technologies such as ET and OPU. There is a significant likelihood that embryos or oocytes obtained from older cows may exhibit lower quality compared to those from younger animals [20]. In the case of *in vitro* fertilization, oocytes with the addition of resveratrol showed a higher rate of successful fertilization [108]. Substances such as N-acetylcysteine and dichloroacetic acid are also utilized [109]. Currently, there is hope centered around the protein Sirtuin 1 (SIRT1). SIRT1 has been detected in mammalian oocytes and embryos (Fig. 3) and is an NAD+-dependent deacetylase. Interestingly, among other promising proteins, it plays a role in regulating cell aging by silencing the information regulator 2-associated enzyme [110]. As oocytes age, the antioxidant response, controlled by SIRT1, decreases. These observations suggest that SIRT1 may be a promising pharmacological agent for improving oocyte quality and *in vitro* fertilization outcomes, especially in the context of aging processes [111, 112]. In human reproductive medicine, ooplasm transfer is well-established in which the ooplasm obtained from a young woman's oocyte donor is transferred to an advanced-age oocyte. Improvement in embryo formation and implantation has been observed. It appears that such a technique could also be effective in cattle. Improvements in embryo formation, implantation, and live birth rate have been observed [113]. The most advanced programs also utilize genomic selection at the embryonic stage through single nucleotide polymorphism (SNP) genotyping and calculation of genomic estimated breeding values (GEBV). Silvestri et al. (2021) suggested that further detailed SNP analysis gathered for GEBV could effectively eliminate aneuploid embryos from the pool, thus improving the number of live births per ET. In their study on 1713 bovine blastocysts (PGT-A method), they found that aneuploid embryos had a 5.8% chance of establishing a pregnancy and a 5.0% chance of resulting in a live birth. In contrast, euploid embryos had a pregnancy establishment rate of 59.6% and a live birth rate of 46.7%. PGT-A improved the overall pregnancy and live birth rates by 7.5% and 5.8%, respectively. It is worth noting that XY embryos exhibited a higher rate of aneuploidy, similar to humans [114]. According to some studies, approximately 40% of in vitro-produced bovine embryos may carry chromosomal abnormalities [115]. However, the use of PGT-A in animal breeding is not yet well-established; it has only recently been adapted for bovine screening studies, yielding promising results [116].

## Summary

Maternal age has important consequences for various aspects of reproduction not only in cattle and women. All the aforementioned factors characterize a key correlation of reproductive aging: as the mother ages, the number of oocytes in her ovaries steadily decreases. This decline plays a crucial role in the context of fertility, as it is an indispensable part of the complex mechanism of fertilization and pregnancy development. The decline in both the number and quality of oocytes is one of the main challenges that affect the reduced ability to conceive in women at a later age. The first factor is the natural consumption of the ovarian reserve, which is already limited from the time of birth of the mother. As the years go by, this process becomes more active and the number of available oocytes steadily decreases. Another important aspect is ovarian atresia, a process of programmed ovarian cell death that takes place in the ovaries from the very beginning of her life. With age, the number of oocytes that undergo this process increases. A decline in oocyte quality is also inherent in the aging process. Studies show that older cows have a lower number of poor-quality oocytes. The oocytes of older animals are more susceptible to genetic mutations and chromosomal aberrations, which increases the risk of poorer-quality embryos. Furthermore, embryo development is strongly influenced by the age of the mother. Older cows show a lower fertilization rate and a higher number of cleaved embryos. In addition, it has been observed that older cattle have relatively less ATP and mDNA than young cattle. The process of ovarian follicle atresia, or programmed ovarian cell death, is more developed in older cows. This phenomenon negatively affects the number of available oocytes. In the context of pregnancy and fetal development, young cows show greater efficiency in conception and better fetal development compared to older animals. There are studies suggesting that a decrease in the production of progesterone, the key hormone responsible for maintaining pregnancy, in older cows may negatively affect pregnancy and the developing fetus. In conclusion, the age of the cattle dam has a significant impact on oocyte quality, embryo development, pregnancy, and the developing fetus. Cattle breeders should take this factor into account in reproduction planning and animal selection. Further research is needed to better understand the mechanisms affecting the reproductive efficiency of cattle at different ages.
